# Robot-assisted vs. laparoscopic nephroureterectomy for upper urinary tract urothelial carcinoma: a systematic review and meta-analysis based on comparative studies

**DOI:** 10.3389/fonc.2022.964256

**Published:** 2022-08-03

**Authors:** Ruoyu Ji, Zhangyuting He, Shiyuan Fang, Wenjie Yang, Mengchao Wei, Jie Dong, Weifeng Xu, Zhigang Ji

**Affiliations:** ^1^ Peking Union Medical College Hospital, Chinese Academy of Medical Sciences and Peking Union Medical College, Beijing, China; ^2^ Department of Urology, Peking Union Medical College Hospital, Beijing, China

**Keywords:** nephroureterectomy, robot-assisted, laparoscopic, urothelial carcinomas, complications, treatment outcome

## Abstract

**Background:**

Robot-assisted nephroureterectomy (RANU) and laparoscopic nephroureterectomy (LNU) are two minimally invasive surgical management for upper urinary tract urothelial carcinomas (UTUC). Though more high-tech, it remains largely unclear whether RANU provides additional benefits over LNU. We aimed to quantitatively compare the perioperative and oncologic outcomes between RANU and LNU.

**Methods:**

The systematic review was performed based on a registered protocol (registration number CRD42022319086). We searched through PubMed, EMBASE and Cochrane databases, as well as conference proceedings and references of review articles (May 2022) for comparative studies reporting perioperative and oncologic outcomes independently in RANU and LNU groups. Selection of studies and data extraction were performed independently by two researchers. Risk of bias was assessed using the modified Newcastle-Ottawa Scale. Results of random-effects meta-analyses were presented as mean differences (MD) or Odds ratio (OR), as appropriate. Subgroup and univariate meta-regression analyses were performed to identify interstudy heterogeneities.

**Results:**

The review included 8470 patients undergoing RANU and 19872 patients undergoing LNU from 12 comparative original studies. RANU was associated with fewer overall complications (OR=0.71, 95%CI: 0.62 to 0.81), longer operative time (MD=27.70, 95%CI: 0.83 to 54.60) and shorter length of stay (MD=-0.53, 95%CI: -0.98 to -0.07) compared to LNU. In addition, patients receiving RANU were more likely to have lymph node dissected (OR=2.61, 95%CI: 1.86 to 3.65). Recurrence and survival outcomes did not differ between two surgical procedures. Sample size, types of LNU and world region were major sources of heterogeneity.

**Conclusion:**

For UTUC patients, RANU offers fewer complications and shorter hospitalization. However, RANU requires longer operative time and shares similar oncologic outcomes compared to LNU. Further randomized designed studies are warranted.

**Systematic Review Registration:**

www.crd.york.ac.uk/prospero/, identifier CRD42022319086.

## Introduction

Radical nephroureterectomy is the gold-standard surgical management for high-risk non-metastatic upper urinary tract carcinomas (UTUC) (1). Over the past 15 years, laparoscopic nephroureterectomy (LNU) has developed rapidly and shares equivalent oncologic outcomes with open nephroureterectomy (2). Further, LNU provides superior performance in reducing blood loss, shortening hospitalization, and accelerating recovery, making it progressively favored than the open procedure (3). However, major concerns regarding LNU have also been raised, especially regarding the extent of lymph node dissection and incidence of port-site metastases (4). Such concerns serve as an impetus to the increasing development of robotic-assisted nephroureterectomy (RANU) (5). The robotic wrists provide extra degrees of freedom for easier distal ureter isolation and bladder closure, and its three dimensional magnified vision helps dissect lymph nodes around great vessels (6). During the last two decades, robotic-assisted surgeries have been increasingly utilized in renal cell carcinoma and prostate cancer (7, 8). However, evidence focusing on RANU in UTUC are mostly limited to observational studies with mixed results. A published meta-analysis containing 87,000 cases compared the perioperative and oncologic outcomes between RANU and LNU (5), but its large proportion of noncomparative studies may lead to bias in results. This underscores the need to perform a systematic review and meta-analysis for quantitative evaluation of the perioperative and oncologic outcomes between RANU and LNU based on comparative studies.

## Materials and methods

We performed the systematic review based on a protocol with the registration number CRD42022319086 and complied with the Preferred Reporting terms for Systematic Review and Meta-Analysis (PRISMA) statement (9). Reporting items were detailed in the PRISMA checklist (**Supplementary Material**).

The purpose of this review was to compare the perioperative and oncologic outcomes between robot-assisted nephroureterectomy and laparoscopic nephroureterectomy.

### Literature search

We searched through PubMed, EMBASE and Cochrane databases. The search strategy in PubMed was: [(pelvis OR pelvic OR ureteral OR ureter OR urothelial OR upper urinary tract) AND (neoplasm OR cancer OR carcinoma OR malignancy) AND (robot OR robot-assisted OR robotic) AND (laparoscopic OR laparoscopy) AND nephroureterectomy]. The search strategy was adapted for EMBASE and Cochrane databases. We also searched references of review articles for relevant studies. The last search update was May 2022.

### Selection of studies

Studies were selected according to the PICOS (patient, intervention, comparator, outcome, study type) approach. Inclusion criteria were: (P) patients aged over 18 years old with a diagnosis of UTUC; (I) undergoing RANU; (C) undergoing LNU; (O) evaluating at least one of the following outcomes: perioperative outcomes (operative time, blood loss, transfusion rate, perioperative complications, lymph node dissection, bladder cuff management, conversion rate, length of stay (LOS), perioperative mortality, postoperative treatment, *etc*), survival outcomes (overall survival (OS), cancer-specific survival (CSS), recurrence-free survival (RFS), recurrence rate, *etc*), pathological outcomes (positive margin rate, lymph node invasion rate, *etc*); (S) retrospective or prospective human studies. Exclusion criteria included (1): noncomparative studies; (2) grey literature lacking details or peer review; (3) insufficient data for quantitative analyses. We set no limitations on language, publication type or publication date. For studies published by the same institution, only the latest or largest study was included. For studies examining the same database for overlapping periods of time, only the largest one was included. Study selection was conducted by two researchers (RYJ and ZYTH) independently, with disagreements resolved through discussion with senior investigators (WFX and ZGJ).

### Data extraction

We extract the following data: (1) study information including publication (article title, authors, year, journal title), study design (patient inclusion and exclusion criteria, grouping and the sample size of each, follow-up duration) and bias control. (2) baseline characteristics including demographics information [age, sex, race, country or region, Charlson comorbidity index (CCI)], tumor location, pre-operative hydronephrosis) and preoperative treatment. (3) perioperative outcomes (operative time, blood loss, transfusion, complications, conversion (open bladder cuff excision is not included), LOS, perioperative mortality, bladder cuff and lymph node management); (4) survival outcomes (OS, CSS, RFS, recurrence and metastasis, postoperative chemotherapy); (5) pathological outcomes (positive margin, lymph node and lymphovascular invasion, grading and staging). We made full use of available materials for data extraction. If required information was not clearly or completely recorded, we contacted the corresponding author and co-authors *via* e-mail. Data extraction was conducted by two researchers (RYJ and ZYTH) independently, with disagreements resolved through discussion with senior investigators (WFX and ZGJ).

### Risk of bias assessment

We assessed the risk of bias using a modified Newcastle-Ottawa Scale (NOS) (10) with the intention of best evaluating our phenomenon of interest (Supplementary Material). The risk of bias was evaluated from three domains: selection, comparability and exposure (or outcome), and each study was awarded with a maximum of 10 scores. A total score of 5 or less, 6-7 and 8 or more was considered low, moderate and high quality, respectively. Risk of bias assessment was conducted by two researchers (RYJ and YTHZ) independently, with disagreements resolved through discussion with senior investigators (WFX and ZGJ).

### Statistical analysis

Basic characteristics of included studies were firstly tabulated. Variables reported by three or more studies were evaluated through quantitative analyses. For continuous data, the mean differences [MD] with 95% confidence intervals [CI] were calculated as the effect measurements. If data were reported as the median with interquartile range, we converted them into the mean with standard deviation through a recommended formula (11). For binary data, the odds ratio (OR) and 95% CI were calculated as the effect measurements. Considering large interstudy heterogeneity, we utilized the random-effects model for quantitative analyses. Heterogeneity across studies were evaluated using the Cochrane chi-square (χ2) and quantified with the *I^2^* statistics. *I^2^* values of 25, 50 and 75% represented low, moderate and high heterogeneity, respectively (12). Publication bias of variables reported by ten or more studies was statistically examined by Egger’s test (13). We performed the following subgroup analyses to explore sources of heterogeneity: mean age, female proportion, world region, sample size and types of LNU (hand-assisted versus standard). Univariate meta-regression analyses were further performed to identify heterogeneity sources across studies. Multivariate meta-regression analyses were not performed due to limited number of studies. All analyses except Egger’s test and meta-regression analyses were performed using Review Manager 5.3.3 (Nordic Cochrane Centre, Copenhagen, Denmark) and STATA version 16 (StataCorp, College Station, TX) was used for Egger’s test and meta-regression analyses. P <0.05 was considered statistically significant.

## Results

### Baseline characteristics

**Figure 1:**
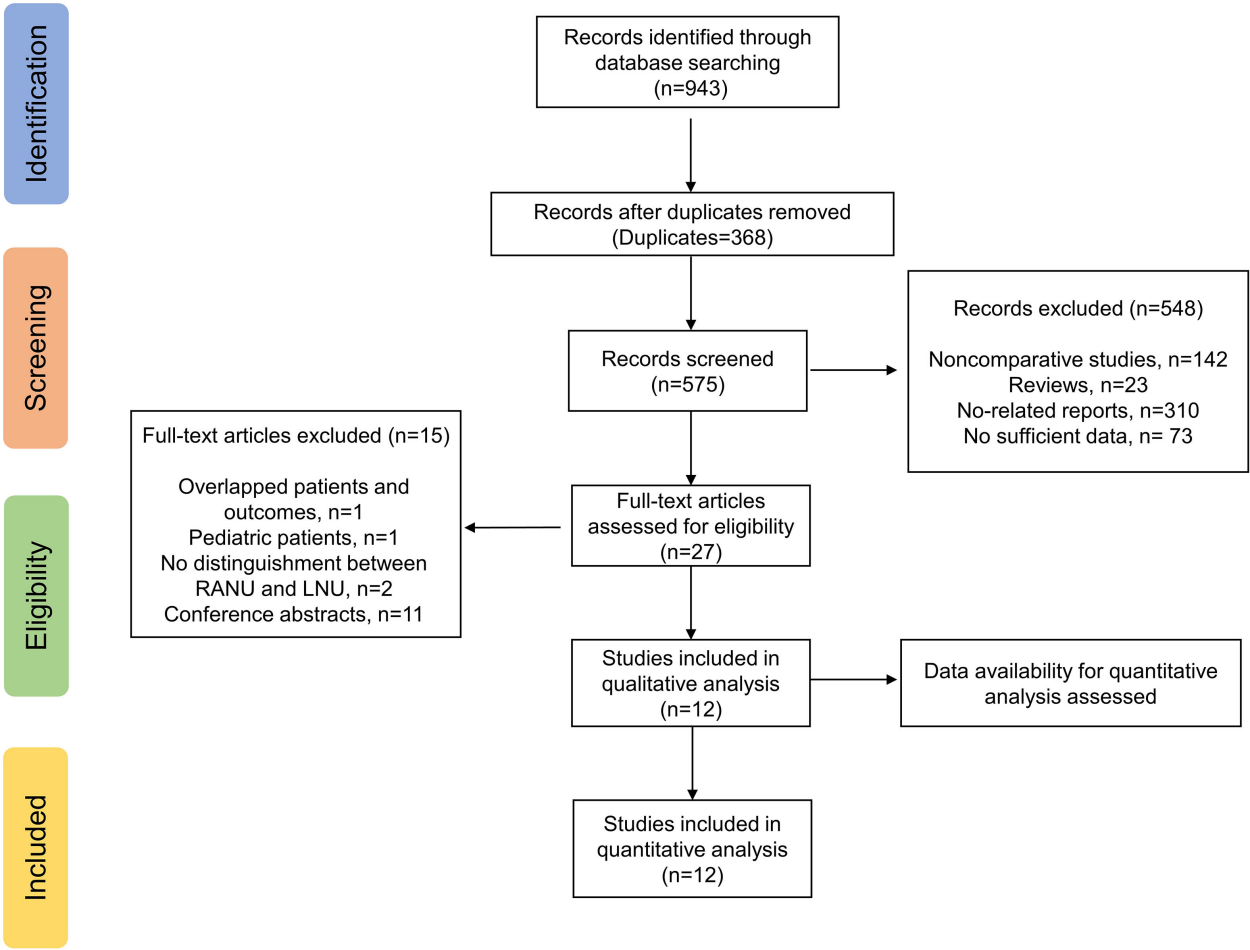
Study flow chart.

The electronic search yielded a total of 943 potentially relevant studies (**Figure 1**). All records were imported into the Endnote with 368 duplicates removed. After evaluating the eligibility of the studies by reading the titles and abstracts, 548 studies were further eliminated. Among the remaining 27 studies, eleven conference abstracts were further excluded (14–24). Two studies (25, 26) shared overlapped patients and outcomes, and therefore the study with fewer patients and published earlier were excluded (26). One study based on pediatric population (27) and two studies which did not distinguish RANU and LNU groups (28, 29) were also excluded. Therefore, a total of twelve original articles (25, 30–40) were ultimately enrolled in quantitative analyses.

All included studies were retrospective comparable studies with a median NOS score of 7 (Range: 6–9) (**Table 1**). Altogether, 8470 patients undergoing RANU and 19872 patients undergoing LNU were included. There were no statistically significant differences between RANU and LNU in basic demographic characteristics including age, sex, ethnicity, pre-operative hydronephrosis and CCI (**Table 2**). However, the RANU group had a significantly shorter follow-up period compared with LNU group (MD = -17.11, 95%CI: -24.52 to -9.71, P<0.001). Also, no significant differences were found with respect to tumor characteristics including tumor location, proportion of staging ≥pT3, high grade, lymph node invasion and lymphovascular invasion between two groups (**Table 2**).

**Table 1:** Basic characteristics of included studies.

Studies	Study design	Mean follow-up time (months)	RANU (n)	LNU (n)	Country/Region	Female proportion (%)	Mean age (years)	Proportion of BCE in RANU (%)	Intracorporeal BCE in RANU (%)	Proportion of BCE in LNU (%)	Intracorporeal BCE in LNU (%)	#NOS
Ambani 2013 [25]	Retrospective	NA	22	22	USA	31.8	70.5 ± 2.2	100	100	90.9	70.0	9
Tinay 2015 [30]	Retrospective	NA	3774	13317	USA	41.9	NA	NA	NA	NA	NA	7
Autorino 2022 [31]	Retrospective	NA	185	91	Global	41.3	71.4 ± 2.5	81.9	NA	63.7	NA	7
Hu 2015 [32]	Retrospective	38.4	18	18	Taiwan	72.2	70.0 ± 6.0	100	100	100	0	7
Byun 2018 [33]	Retrospective	31.3	124	137	Korea	30.3	68.1 ± 10.9	100	NA	100	0	7
Matin 2015 [34]	Retrospective	23.1	37	63	USA, Brazil	38.0	NA	100	100	100	NA	7
Margulis 2020 [35]	Retrospective	NA	1129	1502	USA	37.3	NA	NA	NA	NA	NA	7
Yang 2021 [36]	Retrospective	15.9	10	19	Taiwan	62.1	63.0 ± 8.5	NA	NA	NA	NA	7
Trudeau 2014 [37]	Retrospective	NA	715	735	USA	38.6	71.1 ± 11.2	NA	NA	NA	NA	7
Li 2021 [38]	Retrospective	NA	141	1199	Taiwan	56.9	NA	84.4	NA	84.1	NA	8
Pearce 2016 [39]	Retrospective	NA	2286	2638	USA	NA	NA	NA	NA	NA	NA	6
Ye 2019 [40]	Retrospective	38.8	29	131	China	35.6	64.9 ± 28.6	100	100	100	50.4	7

RANU, robot-assisted nephroureterectomy; LNU, laparoscopic nephroureterectomy; BCE, bladder cuff excision; #Scored by a modified Newcastle-Ottawa Scale with a maximum score of 10. NA, not accessible.

**Table 2:** Comparison of patient and tumor characteristics.

Variables		#RANU vs LNU	*I^2^* (%)	P
Age MD (95% CI), year		0.56 [-0.26, 1.38]	35	0.18
Female proportion OR (95% CI)		1.04 [0.84, 1.28]	74	0.75
Non-white proportion OR (95% CI)		0.88 [0.66, 1.17]	70	0.37
Charlson comorbidity index MD (95% CI)		-0.16 [-0.37, 0.04]	92	0.12
Pre-operative hydronephrosis OR (95% CI)		0.75 [0.48, 1.18]	57	0.21
Follow-up period MD (95% CI), months		-17.11 [-24.52, -9.71]	97	<0.01
Neoadjuvant chemotherapy OR (95% CI)		1.28 [0.73, 2.23]	42	0.39
≥pT3 OR (95% CI)		0.86 [0.74, 1.00]	0	0.05
High grade OR (95% CI)		1.03 [0.81, 1.31]	24	0.82
Lymph node invasion OR (95% CI)		0.82 [0.50, 1.34]	27	0.44
Lymphovascular invasion OR (95% CI)		0.93 [0.69, 1.26]	0	0.64
Tumor location	renal pelvis	1.14 [0.91, 1.42]	0	0.25
	ureter	0.82 [0.64, 1.04]	0	0.10
	both	1.11 [0.78, 1.57]	0	0.57

RANU, robot-assisted nephroureterectomy; LNU, laparoscopic nephroureterectomy; MD, mean differences; OR, odds ratio; CI, confidence interval; #A positive MD or OR favors RANU group.

### Perioperative outcomes

**Figure 2:**
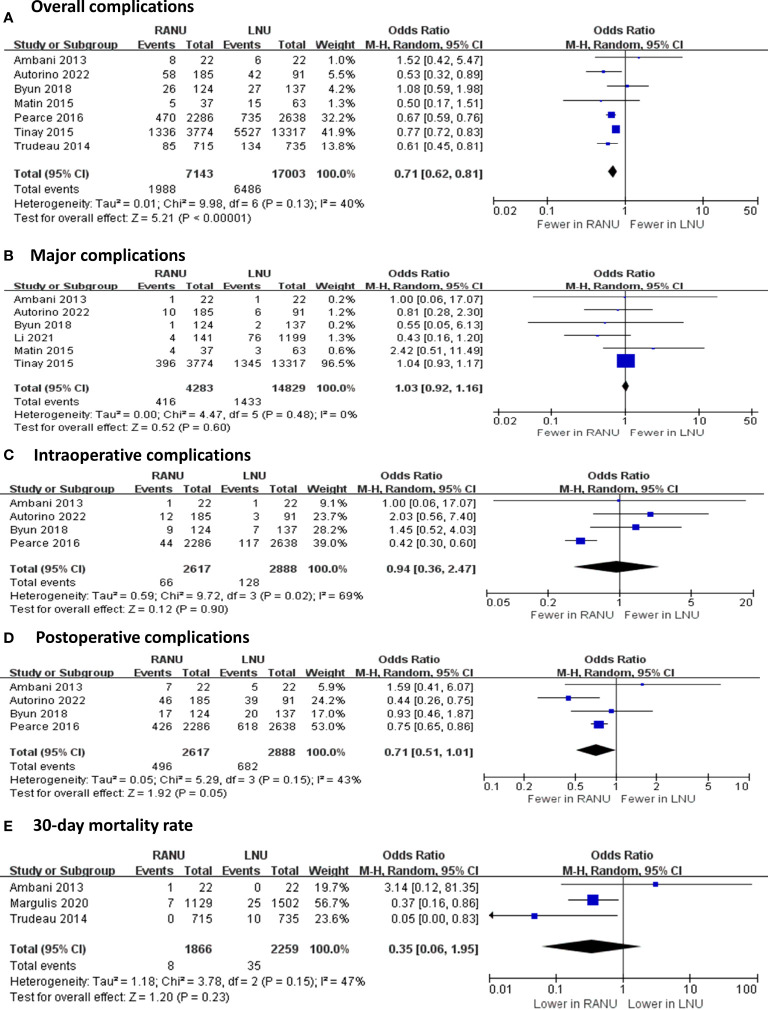
Forest plots of perioperative safety outcomes including overall complications **(A)**, major complications **(B)**, intraoperative complications **(C)**, postoperative complications **(D)** and 30-day mortality rate **(E)** for robotic-assisted nephroureterectomy (RANU) versus laparoscopic nephroureterectomy (LNU).

**Figure 3:**
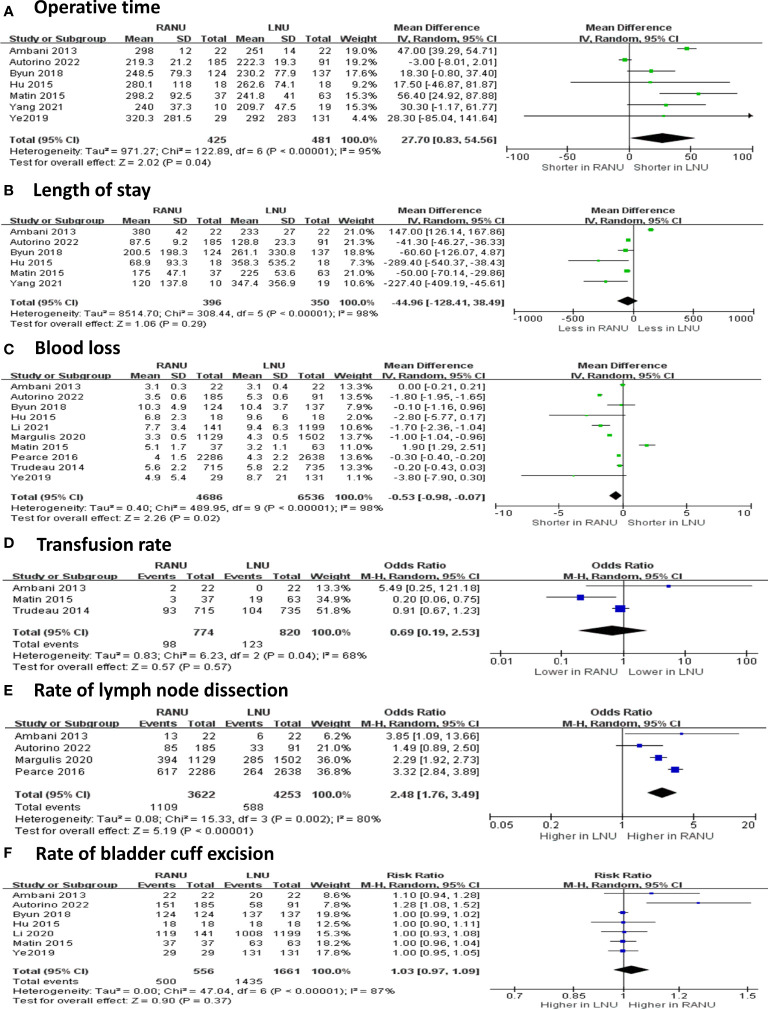
Forest plots of perioperative effectiveness outcomes including operative time **(A)**, blood loss **(B)**, length of stay **(C)**, transfusion rate **(D)**, rate of lymph node dissection **(E)** and rate of bladder cuff excision **(F)** for robotic-assisted nephroureterectomy (RANU) versus laparoscopic nephroureterectomy (LNU).

Regarding operative variable (**Figures 2**, **3**), the RANU group had significantly longer operative time compared with the LNU group (MD=27.70, 95%CI: 0.83 to 54.60), P=0.004). Also, patients undergoing RANU were more likely to receive lymph node dissection compared with patients undergoing LNU (OR=2.48, 95%CI: 1.76 to 3.49, P<0.001). We detected no significant differences in blood loss (MD=-44.96, 95%CI: -128.41 to 38.49, P=0.29), transfusion rate (OR=0.69, 95%CI: 0.19 to 2.53, P=0.57) and rate of bladder cuff excision (OR=1.03, 95%CI: 0.97 to 1.09, P=0.37) between two groups. In terms of surgical complications, RANU was associated with fewer overall complications than LNU (OR=0.71, 95%CI: 0.62 to 0.81, P<0.001). Nevertheless, frequencies of major (Clavien-Dindo≥3) complications (OR=1.03, 95%CI: 0.92 to 1.16, P=0.59), intraoperative complications (OR=0.94, 95%CI: 0.36 to 2.47, P=0.90), postoperative complications (OR=0.71, 95%CI: 0.51 to 1.01, P=0.05) as well as 30-day mortality rate (OR=0.35, 95%CI: 0.06 to 1.95, P=0.23) did not differ significantly between RANU and LNU. Patients undergoing RANU had significantly shorter LOS (MD=-0.53, 95%CI: -0.98 to -0.07, P=0.02) than patients undergoing LNU.

### Oncologic outcomes

**Figure 4:**
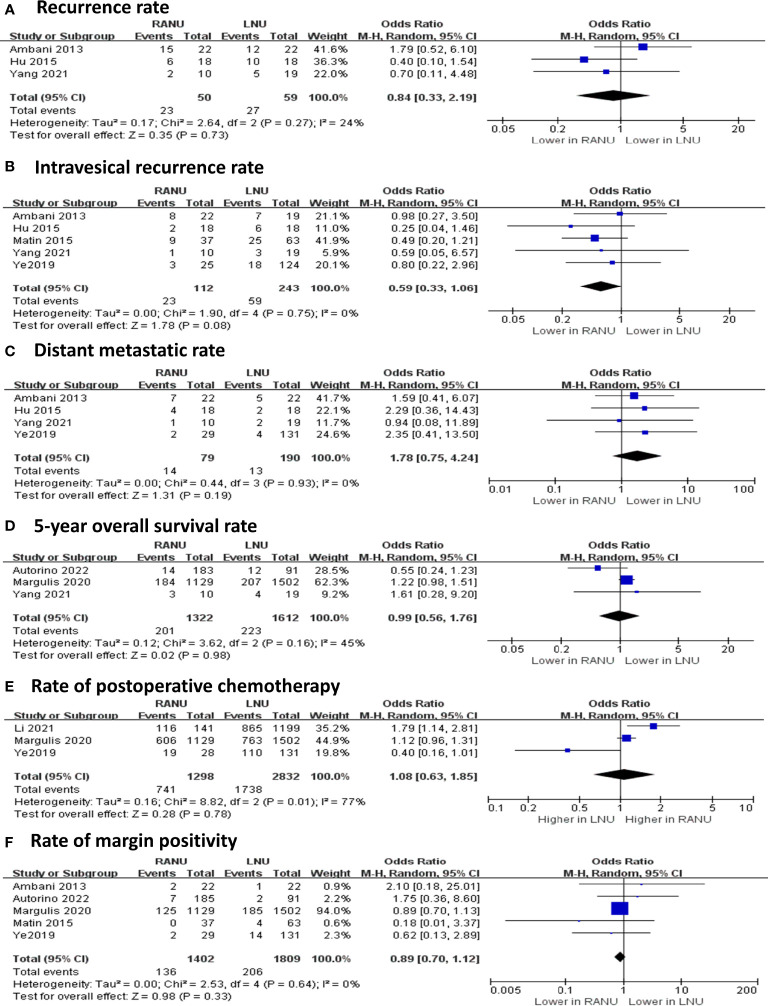
Forest plots of oncologic outcomes including recurrence rate **(A)**, intravesical recurrence rate **(B)**, distant metastatic rate **(C)**, 5-year overall survival rate **(D)**, rate of postoperative chemotherapy **(E)** and rate of margin positivity **(F)** for robotic-assisted nephroureterectomy (RANU) versus laparoscopic nephroureterectomy (LNU).

The two surgical procedures shared similar rate of margin positivity (OR=0.89, 95%CI: 0.70 to 1.12, P=0.33) (**Figure 4**). Also, similar proportions of patients received postoperative chemotherapy in both groups (OR=0.99, 95%CI: 0.56 to 1.76, P=0.98). Regarding recurrence and metastasis, RANU and LNU groups had comparable overall recurrence rate (OR=0.84, 95%CI: 0.33 to 2.19, P=0.73), intravesical recurrence rate (OR=0.59, 95%CI: 0.33 to 1.06, P=0.08) as well as distant metastatic rate (OR=1.78, 95%CI: 0.75 to 4.24, P=0.19). Regarding the long-term survival outcome, the two groups shared similar 5-year OS rate (OR=1.08, 95%CI: 0.63 to 1.85, P=0.78).

### Subgroup analyses and meta-regression analyses

Thresholds for grouping were determined based on median age, median female proportion of overall enrolled patients, median sample size, world region and types of LNU (hand-assisted versus standard) of included studies.

Results of subgroup analyses and univariate meta-regression analyses indicated that mean age, female proportion, world region, sample size and types of LNU all contributed to heterogeneities across studies to varying degrees (**Table 3**).

**Table 3:** Subgroup analyses and univariate meta-regression analyses of perioperative and oncologic outcomes for RANU and LNU.

Group	Subgroups	Studies (n)	^#^MD/OR [95% CI]	*I^2^* (%)	^&^ *I^2^*_sub_ (%)	^*^P
**Overall complications**						
Age	Mean age <70 years	1	1.08 [0.59, 1.98]	–	64	0.10
	Mean age ≥70 years	3	0.61 [0.46, 0.82]	11		
Sex	Female proportion <40%	3	0.98 [0.60, 1.59]	0	19	0.27
	Female proportion ≥40%	2	0.70 [0.51, 0.96]	49		
Sample size	Sample size <250	2	0.83 [0.28, 2.47]	40	0	0.77
	Sample size ≥250	5	0.71 [0.62, 0.81]	52		
Country/region	Asian	1	1.08 [0.59, 1.98]	–	44	0.18
	Non-Asian	5	0.71 [0.63, 0.81]	42		
**Major (Clavien-Dindo≥3) complications**						
Age	Mean age <70 years	1	0.55 [0.05, 6.13]	–	0	0.76
	Mean age ≥70 years	2	0.83 [0.31, 2.21]	0		
Sex	Female proportion <40%	3	1.45 [0.44, 4.75]	0	0	0.57
	Female proportion ≥40%	3	0.89 [0.26, 2.98]	41		
Sample size	Sample size <250	2	1.98 [0.50, 7.73]	9	4	0.31
	Sample size ≥250	4	0.96 [0.73, 1.26]	0		
Country/region	Asian	2	0.45 [0.17, 1.15]	0	68	0.08
	Non-Asian	3	1.05 [0.93, 1.18]	0		
**Intraoperative complications**						
Age	Mean age<70 years	1	1.45 [0.52, 4.03]	–	0	0.79
	Mean age ≥70 years	2	1.80 [0.56, 5.83]	0		
Sex	Female proportion <40%	2	1.39 [0.53, 3.63]	0	0	0.64
	Female proportion ≥40%	1	2.03 [0.56, 7.40]	–		
Sample size	Sample size <250	1	1.00 [0.06, 17.07]	–	0	0.98
	Sample size ≥250	3	0.95 [0.32, 2.84]	72		
Country/region	Asian	1	1.45 [0.52, 4.03]	–	80	0.03
	Non-Asian	2	0.43 [0.30, 0.61]	0		
**Postoperative complications**						
Age	Mean age <70 years	1	0.93 [0.46, 1.87]	–	0	0.72
	Mean age ≥70 years	2	0.72 [0.21, 2.42]	67		
Sex	Female proportion <40%	2	1.04 [0.56, 1.93]	0	75	0.05
	Female proportion ≥40%	1	0.46 [0.26, 0.76]	–		
Sample size	Sample size <250	1	1.59 [0.41, 6.07]	–	30	0.23
	Sample size ≥250	3	0.68 [0.48, 0.96]	50		
Country/region	Asian	1	0.93 [0.46, 1.87]	0	0	0.72
	Non-Asian	2	0.80 [0.53, 1.22]	16		
**30-day mortality**						
Sample size	Sample size <250	1	0.35 [0.06, 1.95]	–	51	0.16
	Sample size ≥250	2	0.21 [0.03, 1.35]	49		
**Operative time**						
Age	Mean age <70 years	3	21.67 [5.50, 37.83]	0	0	0.99
	Mean age ≥70 years	2	21.91 [-27.09, 70.91]	99		
Sex	Female proportion <40%	3	32.89 [16.36, 49.41]	72	37	0.21
	Female proportion ≥40%	3	9.88 [-21.91, 41.66]	76		
Sample size	Sample size <250	4	37.87 [24.67, 51.07]	48	86	0.009
	Sample size ≥250	2	5.57 [-14.90, 26.05]	78		
Types of LNU	Hand-assisted LNU	2	27.83 [-0.44, 56.11]	0	0	0.98
	Standard LNU	5	28.47 [-3.40, 60.34]	97		
Country/region	Asian	3	21.67 [5.50, 37.83]	0	0	0.91
	Non-Asian	3	24.03 [-11.73, 59.79]	98		
**Blood loss**						
Age	Mean age <70 years	2	-121.56 [-278.99, 35.87]	100	50	0.16
	Mean age ≥70 years	2	52.57 [-131.96, 237.10]	65		
Sex	Female proportion <40%	3	13.44 [-139.11, 165.98]	99	9	0.30
	Female proportion ≥40%	2	-111.25 [-287.92, 65.41]	75		
Sample size	Sample size <250	3	-23.08 [-191.15, 145.00]	99	0	0.83
	Sample size ≥250	2	-41.41 [-46.36, -36.46]	0		
Types of LNU	Hand-assisted LNU	2	-248.74 [-395.96, -101.51]	0	87	0.005
	Standard LNU	4	0.23 [-91.29, 91.76]	99		
Country/region	Asian	2	-121.56 [-278.99, 35.87]	65	44	0.18
	Non-Asian	3	48.48 [-144.58, 241.54]	99		
**Length of stay**						
Age	Mean age <70 years	2	-1.53 [-5.21, 2.15]	69	0	0.66
	Mean age ≥70 years	3	-0.67 [-1.90, 0.56]	99		
Sex	Female proportion <40%	6	-0.07 [-0.79, 0.64]	98	95	<0.001
	Female proportion ≥40%	2	-1.80 [-1.94, -1.65]	0		
Sample size	Sample size <250	3	0.34 [-1.50, 2.18]	99	37	0.21
	Sample size ≥250	6	-0.88 [-1.36, -0.40]	98		
Types of LNU	Hand-assisted LNU	3	-2.02 [-2.73, -1.32]	0	83	0.02
	Standard LNU	5	-0.26 [-1.50, 0.98]	99		
Country/region	Asian	4	-1.28 [-2.79, 0.23]	75	57	0.13
	Non-Asian	5	0.10 [-0.83, 1.02]	100		
**Transfusion rate**						
Sample size	Sample size <250	2	0.78 [0.03, 18.83]	73	0	0.92
	Sample size ≥250	1	0.91 [0.67, 1.23]	–		
**Recurrence rate**						
Age	Mean age <70 years	1	0.70 [0.11, 4.48]	–	0	0.86
	Mean age ≥70 years	2	0.87 [0.20, 3.76]	61		
Sex	Female proportion <40%	1	1.79 [0.52, 6.10]	–	59	0.12
	Female proportion ≥40%	2	0.49 [0.16, 1.45]	0		
Country/region	Asian	2	0.49 [0.16, 1.45]	0	59	0.12
	Non-Asian	1	1.79 [0.52, 6.10]	–		
Types of LNU	Hand-assisted LNU	2	0.49 [0.16, 1.45]	0	59	0.12
	Standard LNU	1	1.79 [0.52, 6.10]	–		
**Intravesical recurrence rate**						
Age	Mean age <70 years	2	0.75 [0.24, 2.36]	0	38	0.20
	Mean age ≥70 years	2	0.25 [0.07, 0.87]	0		
Sex	Female proportion <40%	3	0.66 [0.35, 1.25]	0	0	0.40
	Female proportion ≥40%	2	0.34 [0.08, 1.41]	0		
Types of LNU	Hand-assisted LNU	3	0.66 [0.35, 1.25]	0	0	0.40
	Standard LNU	2	0.34 [0.08, 1.41]	0		
Country/region	Asian	3	0.54 [0.21, 1.42]	0	0	0.83
	Non-Asian	2	0.62 [0.29, 1.29]	0		
**Distant metastasis rate**						
Age	Mean age <70 years	2	1.75 [0.42, 7.38]	0	0	0.98
	Mean age ≥70 years	2	1.80 [0.61, 5.33]	0		
Sex	Female proportion <40%	2	1.84 [0.63, 5.32]	0	0	0.93
	Female proportion ≥40%	2	1.68 [0.38, 7.47]	0		
Types of LNU	Hand-assisted LNU	2	1.84 [0.63, 5.32]	0	0	0.93
	Standard LNU	2	1.68 [0.38, 7.47]	0		
Country/region	Asian	3	1.94 [0.62, 6.02]	0	0	0.82
	Non-Asian	1	1.59 [0.41, 6.07]	–		
**Postoperative chemotherapy**						
Age	Mean age <70 years	1	1.61 [0.28, 9.20]	–	17	0.27
	Mean age ≥70 years	1	0.55 [0.24, 1.23]	–		
Sex	Female proportion <40%	1	1.22 [0.98, 1.51]	–	57	0.13
	Female proportion ≥40%	2	0.67 [0.32, 1.40]	17		
Types of LNU	Hand-assisted LNU	1	1.61 [0.28, 9.20]	–	17	0.27
	Standard LNU	1	0.55 [0.24, 1.23]	–		
Country/region	Asian	1	1.61 [0.28, 9.20]	–	0	0.76
	Non-Asian	1	1.22 [0.98, 1.51]	–		
**Rate of margin positivity**						
Sex	Female proportion <40%	4	0.88 [0.69, 1.11]	0	0	0.40
	Female proportion ≥40%	1	1.75 [0.36, 8.60]	–		
Sample size	Sample size <250	4	0.67 [0.20, 2.21]	0	0	0.66
	Sample size ≥250	2	0.90 [0.71, 1.14]	0		
Country/region	Asian	1	0.62 [0.13, 2.89]	–	0	0.65
	Non-Asian	3	0.88 [0.70, 1.12]	0		
**Lymph node dissection**						
Sex	Female proportion <40%	2	2.31 [1.94, 2.76]	0	59	0.12
	Female proportion ≥40%	1	1.49 [0.89, 2.50]	–		
Sample size	Sample size <250	1	3.85 [1.09, 13.66]	–	0	0.48
	Sample size ≥250	3	2.40 [1.67, 3.46]	87		

RANU, robot-assisted nephroureterectomy; LNU, laparoscopic nephroureterectomy; MD, mean differences; OR, odds ratio; CI, confidence interval; #A positive MD or OR favors RANU group; & Heterogeneity across subgroups; *P value of univariate meta-regression analyses which tests for subgroup differences.

World region was the major source of heterogeneity for intraoperative complications. In the non-Asian subgroup, patients undergoing RANU experienced fewer intraoperative complications than patients undergoing LNU (OR=0.43, 95%CI: 0.30 to 0.61), while no significant difference was identified in the Asian subgroup (OR=1.45, 95%CI: 0.52 to 4.30)

Sample size contributed to interstudy heterogeneity for OT. In the small sample size subgroup (sample size<250), the OT for RANU was significantly longer than that for LNU (MD=37.87; 95%CI: 24.67 to 51.07), but the OT was comparable between both procedures in the large sample size subgroup (sample size≥250; MD=9.88; 95%CI: -21.91 to 41.66).

Types of LNU was the major source of heterogeneity for blood loss. The RANU led to significantly less blood loss than hand-assisted LNU (MD=-248.74; 95%CI: -395.96 to -101.51), but was comparable to standard LNU (MD=0.23; 95%CI: -91.29 to 91.76).

Female proportion and types of LNU were major sources of heterogeneity for LOS. The LOS was shorter for RNU in the subgroup having a higher female proportion (≥40%; MD=-1.80; 95%CI: -1.94 to -1.65) as well as when compared with hand-assisted LNU (MD=-2.02; 95%CI: -2.73 to -1.32), but not in the subgroup having a lower female proportion (<40%; MD=-0.07; 95%CI: -0.79 to 0.64) or when compared with standard LNU (MD=-0.26; 95%CI: -1.50 to 0.98).

### Publication bias

The Egger’s test was performed for variables reported by ten or more studies. Egger’s test suggested no publication bias for length of stay (P=0.818).

## Discussion

Despite the increasing use of RANU for UTUC, its pros and cons compared with LNU was poorly evaluated. In this meta-analysis, we identified the differences in perioperative and oncologic outcomes between RANU and LNU based on 12 comparative studies with a total of 28,612 patients. Results demonstrated that RANU, though demanding for longer operative time, was associated with fewer overall complications and shorter LOS compared to LNU. In addition, patients receiving RANU were more likely to have lymph node dissected. Remarkably, RANU provided the above-mentioned advantages without compromising oncologic outcomes.

The prolonged operative time of RANU could be attributed to several factors. First, the Da Vinci Standard/S/Si platform requires robotic re-docking and/or patient repositioning (31), which significantly prolongs the operative time. It is reported that compared to the Da Vinci Xi platform, the Si platform prolongs the operative time by fifty minutes (41, 42). An updated subgroup analysis is warranted when more studies based on the Xi platform are published. Second, our study showed that surgeons were more inclined to perform lymph node dissection in RANU than in LNU, which might prolong operative time in RANU. Future patient-level studies would allow address this confounder. The third possible reason could be the publications of RANU in its early stages (30). The first decade into the 21^st^ century was a rapidly evolving time for robotic-assisted surgeries, and every advanced medical center was just getting started with this new technology (30, 35). As a portion of our studies came from that era, this may prolong the operative time in RANU. Once more medical centers have had experiences with robotic surgeries, the operative time is expected to be shortened. Interestingly, previous literature reported that in total mesorectal excision, prior experience in laparoscopic rectal surgery shortened the learning process of robotic-assisted total mesorectal excision (43). Therefore, more enhanced, structuralized training modalities of robot-assisted urologic surgery await further development, especially for young, inexperienced surgeons (44).

Despite sharing the minimally invasive nature, our analyses demonstrated that RANU was further correlated with a decreased risk of overall complications (OR=0.71, 95%CI: 0.62 to 0.81) when compared to LNU. The result exhibited a low to moderate interstudy heterogeneity (I^2^ = 40%) and subgroup analyses were thus performed. Result of the larger sample size subgroup (sample size ≥250; OR=0.74, 95%CI: 0.63 to 0.86) was consistent with the main outcome. The lower complication rate of RANU compared to LNU owes to its better vision, precision and maneuverability of robotic assistance (45). The robotic arms apply delicate traction force on surrounding vessels and tissues, minimizing complication risks.

In addition, RANU also achieved shorter LOS compared with LNU. This is partially due to its lower complication rates, as intraoperative or postoperative complications significantly prolong hospitalization. Interestingly, subgroup analysis indicated that LOS was comparable in the subgroup with lower female proportion (OR=-0.07, 95%CI: -0.79 to 0.64) and the standard LNU subgroup (OR=-0.26, 95%CI: -1.50 to 0.98). The major heterogeneity for this originated from one study (34), in which robotic group had significantly longer LOS (5.1 vs 3.1). The potential reason, as explained by authors, was that intravesical chemotherapy administrated in postoperative day 4-6 in the RANU group might attenuate the motivation of patients being discharged with a catheter who would have had to return for an outpatient appointment. We conducted a sensitivity analysis by excluding this study, the heterogeneity across hand-assited and standard LNU subgroups became non-significant (I^2^ = 65%, P=0.09). Similar results were also reported in radical and partial nephrectomy (46, 47). A high-quality meta-analysis comparing robotic-assisted radical nephrectomy with laparoscopic radical nephrectomy illustrated that robotic surgeries shortened hospitalization for 0.8 day (46). However, this study proposed that this reduction was mainly caused by hospital volume levels rather than surgical approach (46, 47). This supposition was based on the fact that hospitals equipped with robotics are generally large and pursue high rotation rates, therefore plausible to have shorter LOS. However, our study only included comparable studies, thus eliminating the potential bias brought by hospital volumes. The net reduction of LOS in RANU may thus eventually owes to its lower complication rates.

Our results showed that surgeons were more inclined to perform lymph node dissection in RANU than LNU (OR=2.48, 95%CI: 1.76 to 3.49). Consistently, a retrospective study with 762 RANU and 1,385 LNU cases showed that LNU was associated with lower lymph node dissection rate (48). Though multiple factors contribute to whether a lymph node dissection is performed, including patient demographics, tumor levels, and hospital levels, it is believed that this increase in lymphadenectomy largely came from the improved intracorporeal maneuverability and rotation degrees of robotic arms (48).

Although more lymph node dissections were performed in RANU, our results reported similar recurrence rates (OR=0.99, 95%CI: 0.56 to 1.76, P=0.98) as well as 5-year OS rate (OR=1.08, 95%CI: 0.63 to 1.85, P=0.78) between RANU and LNU, suggesting that surgical approach does not alter oncologic outcomes. Noticeably, the overall survival data available for quantitative analysis was only reported by three enrolled studies (35, 38, 40). Hence, we were unable to conduct comprehensive and stable analyses, and this result should be interpreted with caution. Whether more aggressive lymph node dissection improves prognosis of UTUC remains controversial. Current European Association of Urology guideline recommends a template-based lymph node dissection for all UTUC patients, as this has been proven to improve cancer-specific survival and reduce risks of local recurrence, especially in high-stage disease of the renal pelvis (49). On the contrary, a recent result from the robotic surgery for upper tract urothelial cancer study (ROBUUST) registry found that lymph node dissection during RANU was not associated with improved overall survival, though it may provide prognostic information (50). Future prospective multicenter validation is expected to acquire high-level evidence for this clinical issue.

To the best of our knowledge, the present meta-analysis provides the most updated assessments of current evidence regarding perioperative and oncologic outcomes between RANU and LNU, which are two broadly adopted minimally invasive techniques for UTUC. Despite this, this study has several limitations. Enrolled studies were mostly retrospective and non-randomized, introducing potential bias to the analyses. Selection bias is inherent in studies using administrative data. Moreover, some results reported by enrolled studies could not be utilized for quantitative analyses, limiting the number of studies imported in the model. Several critical survival outcomes such as pooled overall survival, cancer specific survival and progression-free survival could not be compared due to limited studies and distinct reporting forms, hindering a pertinent analysis of comparing long-term efficacy between two procedures. Also, there is moderate to high heterogeneity across studies, even though sources of heterogeneity were partly identified by subgroup analyses, demonstrating the underpowered nature of comparisons. Therefore, with continuous publication of articles, the update of the meta-analysis is still warranted to improve the above deficiencies.

## Conclusions

In summary, our review suggests that for UTUC patients undergoing nephroureterectomy, robot-assisted technique seems to offer some advantages compared to LNU, including fewer overall complications, shorter hospitalization and more dissection of lymph nodes. However, RANU requires longer operative time and shares similar oncologic outcomes compared to LNU. These findings should be interpreted with caution due to substantial interstudy heterogeneity and limited sample size. The update of meta-analysis is warranted with more randomized designed studies performed.

## Data availability statement

The original contributions presented in the study are included in the article/**supplementary material**. Further inquiries can be directed to the corresponding authors.

## Author contributions

WX and ZJ are the guarantor of this research and initiated this study. WX and RJ contributed to the design of the study. RJ, ZH, and SF contributed to the study selection and data extraction. RJ, ZH, WY, and MW contributed to data analyses. RJ and SF drafted the manuscript. JD and WX revised the manuscript. All the authors contributed to result interpretation and final approval of the manuscript.

## Conflict of interest

The authors declare that the research was conducted in the absence of any commercial or financial relationships that could be construed as a potential conflict of interest.

## Publisher’s note

All claims expressed in this article are solely those of the authors and do not necessarily represent those of their affiliated organizations, or those of the publisher, the editors and the reviewers. Any product that may be evaluated in this article, or claim that may be made by its manufacturer, is not guaranteed or endorsed by the publisher.
