# Supplementary material for: Robot-assisted vs. laparoscopic nephroureterectomy for upper urinary tract urothelial carcinoma: a systematic review and meta-analysis based on comparative studies
Source: Front Oncol. 2022 Aug 3;12:964256. doi: 10.3389/fonc.2022.964256 (PMC9382403; doi:10.3389/fonc.2022.964256)
Supplement: Supplementary file 1 [file DataSheet_1.docx]

**NEWCASTLE - OTTAWA QUALITY ASSESSMENT SCALE**

**NON-RANDOMIZED STUDIES**

Note: A study can be awarded a maximum of one star for each numbered item within the Selection and Exposure categories. A maximum of three stars can be given for Comparability.

**Selection**

1) Is the case definition adequate?

a) yes, with independent validation **🟑**

b) yes, eg record linkage or based on self reports

c) no description

2) Representativeness of the cases

a) consecutive or obviously representative series of cases **🟑**

b) potential for selection biases or not stated

3) Selection of Controls

a) drawn from the same source as the case group **🟑**

b) drawn from a different source

c) no description

4) Definition of Controls

a) yes, with independent validation **🟑**

b) yes, eg record linkage or based on self reports

c) no description

**Comparability**

1) #Comparability of cases and controls on the basis of the design or analysis

a) study controls for tumor characteristics including staging, grading, location, lymph node invasion and lymphovascular invasion, *etc***🟑** ****

b) study controls for any demographic characteristics including age, sex, BMI and ethnicity, *etc***🟑**

c) study controls Charlson comorbidity index (CCI) or American Society of Anesthesiologists score (ASA)**🟑**

**Exposure/Outcomes**

#1) Ascertainment of exposure

a) secure record (eg, surgical records or clearly reported databases) **🟑**

b) structured interview where blind to case/control status ****

c) interview not blinded to case/control status

d) written self-report or medical record only

e) no description

2) Same method of ascertainment for cases and controls

a) yes **🟑**

b) no

#3) Follow-up period

a) comparable duration for both groups**🟑**

b) noncomparable duration for both groups

c) no description

Items marked with # were modified with the intention of best evaluating our phenomenon of interest
